# Oncological and Functional Outcome after Surgical Treatment of Early Glottic Carcinoma without Anterior Commissure Involvement

**DOI:** 10.1155/2014/464781

**Published:** 2014-06-02

**Authors:** Jovica Milovanovic, Ana Jotic, Vojko Djukic, Bojan Pavlovic, Aleksandar Trivic, Sanja Krejovic-Trivic, Andjela Milovanovic, Aleksandar Milovanovic, Vera Artiko, Bojan Banko

**Affiliations:** ^1^Medical Faculty Belgrade, University of Belgrade, Belgrade, Serbia; ^2^Clinic for Otorhinolaryngology and Maxillofacial Surgery, Clinical Centre of Serbia, Pasterova 2, Belgrade, Serbia; ^3^Clinic for Medical Rehabilitation, Clinical Center of Serbia, Belgrade, Serbia; ^4^Institute for Occupational Health of Serbia “Dr Dragomir Karajovic,” Belgrade, Serbia; ^5^Institute for Nuclear Medicine, Clinical Centre of Serbia, Belgrade, Serbia; ^6^Center for Radiology and Magnetic Resonance Imaging, Clinical Center of Serbia, Belgrade, Serbia

## Abstract

*Introduction*. Glottic carcinoma can be successfully diagnosed in its early stages and treated with high percentage of success. Organ preservation and optimal functional outcomes could be achieved with wide array of surgical techniques for early glottic cancer, including endoscopic approaches or open laryngeal preserving procedures, making surgery the preferred method of treatment of early glottic carcinoma in the last few years. *Material and Methods*. Prospective study was done on 59 patients treated for Tis and T1a glottic carcinoma over a one-year time period in a tertiary medical center. Patients were treated with endoscopic laser cordectomy (types II–IV cordectomies according to European Laryngological Society classification of endoscopic cordectomies) and open cordectomy through laryngofissure. Follow-up period was 60 months. Clinical and oncological results were followed postoperatively. Voice quality after the treatment was assessed using multidimensional voice analysis 12 months after the treatment. *Results*. There were no significant differences between oncological and functional results among two groups of patients, though complications were more frequent in patients treated with open cordectomy. *Conclusion*. Endoscopic laser surgery should be the first treatment of choice in treatment of early glottic carcinomas, though open approach through laryngofissure should be available for selected cases where anatomical factors present limiting adequate tumor removal.

## 1. Introduction

Glottic carcinoma can be successfully diagnosed in its early stages, with high percentage of success. Treatment options include open cordectomy through laryngofissure, endoscopic cordectomy, or radiotherapy, but treatment guidelines are still based on low-level evidence. Cochrane review concluded that there is currently insufficient evidence to guide management decisions on the most effective treatment [1]. In many European countries, radiotherapy was the first choice of treatment for patients with early glottic cancer, with its high local control rates and satisfactory functional results [2, 3]. Still, choosing the optimal treatment option remains a complicated issue. Factors that influence choice of treatment include tumor characteristics, patient's preference, cost, duration and availability of the treatment, and risk of complications [4]. In the last few years there have been a number of papers challenging the opinion of radiotherapy as modality of treatment with less morbidity and more effectiveness. Organ preservation and optimal functional outcomes could also be achieved with wide array of surgical techniques, including endoscopic approaches or open laryngeal preserving procedures, making surgery good alternative and more preferred treatment choice to radiotherapy [5–7].

The aim of this study was to evaluate oncological and functional results of different surgical treatments for Tis and T1 glottic carcinoma and establish their effectiveness.

## 2. Material and Methods

This prospective study was conducted with 59 patients treated for Tis and T1a glottic carcinoma over a one-year time period (between November 1, 2006, and October 31, 2007) in the Institute of Otorhinolaryngology and Maxillofacial Surgery of the Clinical Centre of Serbia in Belgrade. This study was approved by the Institutional Review Board, and all patients provided their informed consent prior to inclusion in the study. Patients had no previous surgical or radiation treatment for malignancy.

Stroboscopic exam was conducted before surgery and on every follow-up control exam. Stroboscopy was performed with the Storz Endovision Telecam DX 20 Pal and Storz Pulsar 20 (Karl Storz GmbH & Co., Tuttlingen, Germany) during modal pitch at comfortable intensity on sustained vowel /*i*/. The following parameters were noted preoperatively and 12 months postoperatively on the operated vocal fold: (1) glottic occlusion (complete and incomplete), (2) phase symmetry (symmetrical, asymmetrical, or not assessable vocal fold vibration), (3) mucosal wave (normal, decreased, or absent), (4) nonvibratory segment (present, absent, or nonassessable), and (5) ventricular activity (present or absent). Stroboscopic assessment was done by two experienced otorhinolaryngologists.

Mucosal extension, depth of vocal fold infiltration, and size of the tumor were assessed with laryngomicroscopy. Group treated with endoscopic transoral cordectomy involved patients with tumor size up to 10 mm, localized on upper surface or free edge of one vocal fold with preserved mobility, without anterior commissure involvement. Patients with tumor localized on one vocal fold with preserved mobility, tumor diameter greater than 10 mm with deeper infiltration, or inadequate endoscopic tumor exposure due to anatomical limitations were treated with surgical cordectomy.

Endoscopic transoral cordectomy was done in 26 patients (types II–IV cordectomies according to recommended European Laryngological Society classification of endoscopic cordectomies) [8]. Endoscopic cordectomies were conducted with a Sharplan Lumenis 40C CO_2_ laser, with a Carl Zeiss Surgical OPMI Sensera optical microscope and with patients under general endotracheal anesthesia. Open cordectomy was conducted in 33 patients. Patients were staged using the TNM clinical classification of the International Union Against Cancer (UICC, 2002) as TisN0 M0 and T1N0 M0 [9], based on clinical examination, laryngomicroscopy, and pathohistology. The open surgical approach involved laryngofissure with cordectomy in general endotracheal anesthesia. Patients with surgical margins that were positive of malignancy received postoperative radiotherapy and were not included in this study.

Multidimensional voice and speech analysis were performed with Tiger DRS software, preoperative and 12 months after the treatment. Acoustic parameters were determined with Vocal Assessment program by analyzing vocal results of patients pronouncing continuous vocal /*a*/. Fundamental frequency (*F*0, Hz), jitter, shimmer, and normalized noise energy (NNE, dB) were followed.

Follow-up period was 5 years. Patients were examined every month postoperatively during the first year, every three months during the second and third years, and every six months during the fourth and fifth years. Local recurrence was defined as carcinoma occurring after completion of primary treatment independent of the localization in any part of the glottis. Recurrent disease was confirmed by laryngomicroscopy and pathohistological analysis of biopsied lesion.

IBM SPSS Statistics 20 (IBM Corporation, New York, NY) was used for the data analysis. Overall, recurrence-free and disease-specific survival were calculated according to the Kaplan-Meier method; the Log-rank test was used to compare survival parameters between patient groups. Wilcoxon signed-rang test was used to compare stroboscopic parameters before and after the treatment between groups. Student's *t*-test was used to compare mean values for acoustic parameters between patients groups before and after the treatment. *P* values lower than 0.05 were considered statistically significant.

## 3. Results

The study included 53 males (89.8%) and 6 females (10.2%), with an average age of 56.73 years. Endoscopic laser cordectomy was done in 26 patients (24 males and 2 females) and open cordectomy through laryngofissure in 33 patients (29 males and 4 females). Most of the patients were staged as T1aN0 M0 (19 in endoscopic laser cordectomy group and 30 in open cordectomy group). Most of our patients were smokers (94.9%). Recurrent disease was noted in 7.7% of patients treated with endoscopic laser cordectomy and in 9.1% of patients treated with open cordectomy (Table 1).

Before treatment, all patients in both groups were complaining of dysphonia. Considering other symptoms, patients were complaining of pain, cough, dysphagia, and dyspnea, but the percentage of those patients was small in both groups (Table 2).

In endoscopic laser cordectomy group, in Tis patients, cordectomy type II was done in 4 patients and type III in 1 patient. In T1aN0 M0 patients cordectomy type III was done in 15 and type IV in 6 patients (according to recommended ELS classification for endoscopic cordectomies).

Stroboscopic signs described preoperatively and 12 months postoperatively were shown in Table 3. Preoperative stroboscopic signs were similar between groups. There was a significant difference only in the number of patients with a normal mucosal wave between groups (15.4% versus 0%, *P* = 0.047). Postoperative results varied significantly between groups. Comparing the stroboscopic signs between the groups, there were significantly more patients with completely absent mucosal wave (63.6% versus 23.1%, *P* = 0.008) and with nonvibratory segment that could not be assessed (42.4% versus 19.2%, *P* = 0.003) in open cordectomy group. In patients treated with endoscopic laser cordectomy, there were significant changes in phase symmetry (Wilcoxon signed-rang test, *P* = 0.014) and mucosal wave (Wilcoxon signed-rang test, *P* = 0.023) before and 12 months after treatment. In open cordectomy group, significant difference was noted in phase symmetry (Wilcoxon signed-rang test, *P* = 0.001), mucosal wave (Wilcoxon signed-rang test, *P* = 0.00), nonvibratory segment (Wilcoxon signed-rang test, *P* = 0.00), and ventricular activity (Wilcoxon signed-rang test, *P* = 0.008) before and 12 months after treatment.

Average duration of hospitalization for endoscopic laser cordectomy group was 3.3 days and for open cordectomy group 7.5 days. There was a significant difference between groups in postoperative complication occurrence (*χ*² = 5.67, *P* = 0.017). In endoscopic laser cordectomy group, temporary tracheotomy was done postoperatively in only one patient (3.8%). In patients treated with open cordectomy, there was local wound infection in 6.1%. Temporary tracheotomy was done postoperatively in 9.1% of the cases. Postoperatively, subcutaneous emphysema was noted in 6.1% and wound dehiscence in 6.1% of the cases (Table 4).

**Figure 1:**
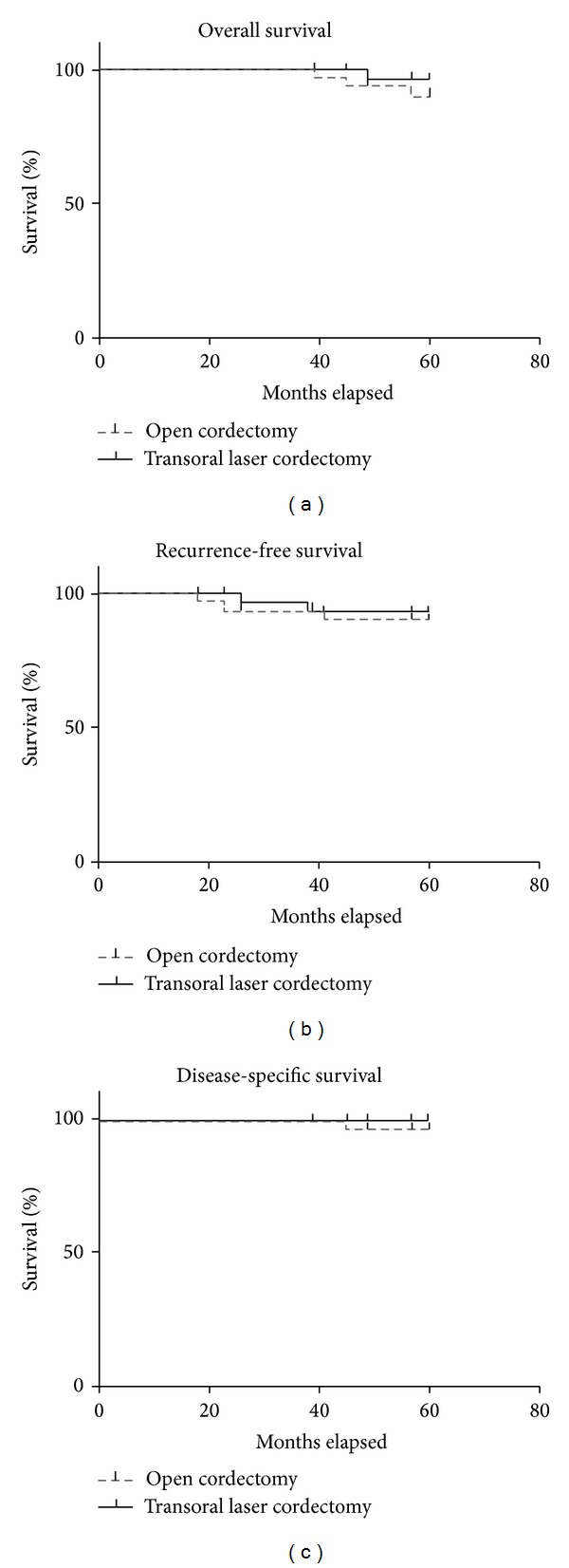
Five-year overall survival and recurrence-free and disease-specific survival.

Five-year overall survival and recurrence-free and disease-specific survival were calculated according to Kaplan-Meier method (Figure 1). The follow-up period for patients was 60 months. Five-year overall survival for open cordectomy patients was 91% and for endoscopic laser cordectomy 96%. The log rank test did not show significant difference between groups (log rank = 0.04; *P* > 0.05). Five patients died during the duration of the study: 3 from cardiovascular diseases and one from pulmonary malignancy and none related with laryngeal malignancy. One patient treated with open cordectomy had anterior commissure recurrence with thyroid cartilage infiltration which required total laryngectomy in further treatment. Postoperatively, radiotherapy was conducted, but 45 months after his first treatment that patient died from laryngeal malignancy. Five-year recurrence-specific survival for open cordectomy patients was 91% and for endoscopic laser cordectomy was 92%, without significant differences between groups (log rank = 0.17; *P* > 0.05). Five-year disease-specific survival for open cordectomy patients was 97% and for endoscopic laser cordectomy was 100%. In three patients with recurrent disease, total laryngectomy was done, mainly due to localization of the recurrent tumor. In these patients, anterior commissure was involved and thyroid cartilage infiltrated, according to control CT scans. Laryngeal preservation was done in two patients, with partial vertical laryngectomies. Deglutition was preserved in all patients. Unilateral resection of ventricular folds which was done in some of the patients treated with types III and IV endoscopic laser cordectomy had no impact on swallowing.

Average values of acoustic parameters with standard deviation before the treatment and 12 months after the treatment are shown in Table 5. Student's *t*-test was used to compare the difference of average values for each parameter between groups. Values of jitter significantly varied between the groups postoperatively (*Z* = 9.941, *P* = 0.003). Comparing mean values of acoustic parameters before and after treatment, in patients treated with endoscopic laser cordectomy, there were significant changes in values of* F*0 (Student's *t*-test, *P* = 0.007), jitter (Student's *t*-test, *P* = 0.00), and shimmer (Student's *t*-test, *P* = 0.001). Jitter (Student's *t*-test, *P* = 0.00) and shimmer (Student's *t*-test, *P* = 0.016) increased significantly postoperatively in patients treated with open cordectomy. NNR increased in both groups, but not significantly.

## 4. Discussion

Conservation surgery (open or endoscopic laser resection) and radiotherapy are valid options for treating T1a glottic lesions, but selection criteria are still subjective [10]. Though radiotherapy is considered the treatment of choice in many European countries, there are a number of recent studies emphasizing the importance of surgery in treatment of early glottic carcinoma [2–4, 11]. Both surgery and radiotherapy provide good locoregional control and satisfactory functional results, so other factors come into consideration when choosing the adequate treatment.

Technical aspects of both treatments are also a consideration. Endoscopic laser cordectomy may not be possible because of patient morphology or comorbidities, or if a surgeon is inexperienced in the technique. Then, open surgery becomes a valid option. Alternatively, the availability and logistics of 6 to 7 weeks of radiotherapy also become a decision-making factor [7]. In countries with poorer health service, facts such as reliability of follow-up, distance from treatment facility, and significant time delay of radiotherapy because of small number of radiology centers must also be considered [4]. Duration of surgical treatment is considerably shorter comparing to radiotherapy and thus preferred by the patients. Also, surgical treatment, especially endoscopic surgery, is proven to be much more cost effective than radiotherapy [12–14]. From an oncological point of view, recurrent disease after radiotherapy must be treated surgically. In those cases, some authors consider that detection of recurred disease is difficult, due to postradiation edema [15]. This makes conservative surgery highly unlikely, and up to 50% of those patients require total laryngectomy due to the progression of the disease [16]. Larynx preservation, disease-specific survival, and overall survival were significantly less favorable in patients treated initially with radiotherapy [17–19]. Functional results after radiotherapy were poorer comparing to those treated with endoscopic laser resection; both perturbation measures (jitter and shimmer) and aerodynamic parameters were more satisfactory in patients after laser surgery [20–22]. All these facts promote surgical management as a preferable treatment of early glottic carcinoma.

Local disease control for surgical treatment of early glottic carcinoma varies in different studies, from 6% to 11.2% [20, 23, 24]. In our study, recurrent malignancy was noted in 7.7% patients in endoscopic laser cordectomy group and in 9.1% in open cordectomy group. Comparing different surgical approaches, Karatzanis et al. had 5-year disease-specific survival of 96.5% for T1a cases and no statistically significant differences noted between different types of procedures: cordectomy and transoral laser microsurgery [11]. Motta et al. published survival rate of 85% and adjusted survival rate of 97% in 432 T1aN0 M0 patients treated with transoral laser microsurgery. If the outcome was lethal, causes of death were other diseases (9.2%), secondary tumor (7.4%), local recurrence (3.5%), nodal recurrence (0.5%), and distant metastasis (0.2%) [25]. In our study cause of death was other diseases in 8.47% cases and local recurrence in 1.7%. Five-year overall survival and recurrence-free and disease-specific survival were over 90% for both groups. Complications were more frequent in patients treated with open cordectomy. de Diego et al. [26] in the series of 104 patients treated with cordectomy through laryngofissure had serohematoma in 26.9%, wound infection in 6.7%, postoperative bleeding in 5.8%, wound dehiscence in 3.8%, and pharyngocutaneous fistula in 1% of the patients.

Regarding stroboscopic evaluation, there is a greater incidence of structural abnormalities (scar tissue, granuloma, and anterior commissure web) and functional problems (incomplete closure, abnormal mucosal wave, and vocal fold immobility) in greater resections than in lesser resections, leaving a bigger portion of functional muscle [7, 21]. Development of a vocal fold scar tissue was described 6 months after surgical injury [27]. Improvements in the amplitude of mucosal wave were visible 6 months after the procedure and continue to improve up to 14 months after the procedure [28]. Our clinical assessments by stroboscopy closely correlated with vocal analysis results. Studies examining the vocal parameters in patients treated with laser cordectomy noted that fundamental frequency (*F*0) tends to be higher in patients for more extended resections [22, 29–31]. Postoperatively, the voice changes significantly because of the removal of the vibrating tissue of the vocal cord in patients treated with open cordectomy and laser cordectomy, with lower mass leading to higher fundamental frequencies. In our study,* F*0 showed a tendency of decreasing postoperatively, but these values were still higher than those in normophonic speakers. Functional outcomes were related to the extent of necessary resection and vibratory pattern of the treated vocal fold detected by stroboscopy.

Jitter and shimmer are acoustic characteristics of voice signal and represent cycle-to-cycle variations of fundamental frequency and waveform amplitude, respectively. Values of jitter and shimmer above a certain threshold are considered being related to pathological voices, usually perceived as breathy, rough, or hoarse voices. Stroboscopy postoperatively indicated that vibration symmetry of treated vocal fold was asymmetric or not assessable in most of the cases. This is probably a result of irregular vibration pattern and patients' inability to compensate newly created incomplete glottis occlusion, confirmed by recorded values of jitter and shimmer which were higher in both groups 12 months after the treatment. Also, we detected a higher number of patients with ventricular activity postoperatively. Motta et al. [25] noted the appearance of nonglottal voicing in patients with type IV cordectomies, which was present in our study; out of 6 patients treated with type IV cordectomy, in 4 of them ventricular activity persisted 12 months postoperatively. This also influenced the values of examined vocal parameters postoperatively. Normalized noise energy (NNE, dB) is a parameter influenced by frequency and amplitude variations and highly sensitive to changes in jitter and shimmer. It was suggested that NNE is extremely helpful in assessing breathy voice [32]. Patients treated with open cordectomy had confirmed incomplete glottic occlusion in more cases than the ones treated with endoscopic laser cordectomy. Values of NNE, which directly correlated with the stroboscopically confirmed degree of incomplete glottic occlusion, improved in both groups 12 months after the treatment, with no significant difference between their values. Number of patients with incomplete glottic occlusion was reduced in both groups postoperatively, according to stroboscopic findings, confirmed by the values of NNE.

Open surgery offers very similar oncological and functional results, but with more probable postoperative complications, of which most are manageable. It should be stressed that open cordectomy through laryngofissure is a valuable option for patients not anatomically suitable for endoscopic surgery or for treatment of recurrent disease. In conclusion, endoscopic laser surgery is highly efficient and low-cost procedure and should be considered and proposed as a first choice treatment for early glottic carcinoma.

## Figures and Tables

**Table 1:** Demographic and clinical variables.

	Endoscopic laser cordectomy	Open cordectomy
Gender *n* (%)		
Male	24 (92.3)	29 (87.9)
Female	2 (7)	4 (12.1)
Age (mean ± SD)	57.65 ± 10.31	55.82 ± 8.78
T stage *n* (%)		
Tis	5 (19.2)	3 (9.1)
T1a	21 (80.8)	30 (90.9)
Smoking *n* (%)		
Yes	25 (96.2)	31 (93.9)
No	1 (3.8)	2 (6.1)
Recurrent carcinoma *n* (%)		
Yes	2 (7.7)	3 (9.1)
No	24 (92.3)	31 (90.9)

**Table 2:** Patients' symptoms in both treated groups.

*n* (%)	Endoscopic laser cordectomy	Open cordectomy
Dysphonia		
Yes	26 (100)	33 (100)
No	0 (0)	0 (0)
Pain		
Yes	2 (7.7)	3 (9.1)
No	24 (92.3)	30 (90.9)
Cough		
Yes	3 (11.5)	4 (12.1)
No	23 (88.5)	29 (87.9)
Dysphagia		
Yes	2 (7.7)	1 (3)
No	24 (92.3)	32 (97)
Dyspnea		
Yes	3 (8.1)	2 (6.1)
No	25 (96.2)	31 (93.9)

**Table 3:** Stroboscopic signs in both groups of patients before treatment and 12 months after the treatment.

Stroboscopic sign	Pretreatment		After 12 months	
	Endoscopic laser cordectomy	Open cordectomy	Endoscopic laser cordectomy	Open cordectomy
Glottic occlusion *n* (%)				
Complete	7 (26.9)	6 (18.2)	10 (38.5)	8 (24.2)
Incomplete	19 (73.1)	27 (81.8)	16 (62.5)	25 (75.8)
	*P = 0.421 *		*P = 0.814 *	
Phase symmetry *n* (%)				
Symmetrical	1 (3.8)	0 (0)	3 (11.5)	0 (0)
Asymmetrical	25 (96.2)	33 (100)	18 (69.2)	22 (66.7)
Not assessable	0 (0)	0 (0)	5 (19.3)	11 (33.3)
	*P = 0.256 *		*P = 0.087 *	
Mucosal wave *n* (%)				
Normal	4 (15.4)	0 (0)	2 (7.7)	0 (0)
Decreased	22 (84.6)	32 (97)	18 (69.2)	12 (36.4)
Absent	0 (0)	1 (3)	6 (23.1)	21 (63.6)
	P = 0.047*		P = 0.008*	
Nonvibratory segment *n* (%)				
Present	18 (69.2)	29 (87.9)	14 (53.9)	19 (57.6)
Absent	8 (30.8)	4 (12.1)	7 (26.9)	0 (0)
Not assessable	0 (0)	0 (0)	5 (19.2)	14 (42.4)
	*P = 0.77 *		P = 0.003*	
Ventricular activity *n* (%)				
Present	2 (7.7)	3 (9.1)	4 (15.4)	10 (30.3)
Absent	24 (92.3)	30 (90.9)	22 (84.6)	23 (69.7)
	*P = 0.848 *		*P = 0.181 *	

*P < 0.05.

**Table 4:** Postoperative complications.

Patient groups	Local infection *n* (%)	Tracheotomy *n* (%)	Emphysema *n* (%)	Wound dehiscence *n* (%)
Endoscopic laser cordectomy	0/26 (0)	1/26 (3.8)	0/26 (0)	0/26 (0)
Open cordectomy	2/33 (6.1)	3/33 (9.1)	2/33 (6.1)	2/33 (6.1)
Total *N* (%)	**2/59 (3.4)**	**4/59 (6.8)**	**2/59 (3.4)**	**2/59 (3.4)**

**Table 5:** Acoustic parameters in both groups of patients before treatment and 12 months after the treatment.

	Pretreatment (mean ± SD)		After 12 months (mean ± SD)	
	Endoscopic laser cordectomy	Open cordectomy	Endoscopic laser cordectomy	Open cordectomy
*F*0 (Hz)	149.49 ± 21.78	149.62 ± 28.2	144.32 ± 23.1	146.66 ± 26.69
Student's *t*-test	*P = 0.399 *		*P = 0.757 *	
Jitter (%)	0.52 ± 0.15	0.47 ± 0.15	0.71 ± 0.27	0.56 ± 0.16
Student's *t*-test	*P = 0.684 *		P = 0.003*	
Shimmer (%)	5.78 ± 0.98	5.6 ± 0.97	6.47 ± 1.22	6.02 ± 1.04
Student's *t*-test	*P = 0.341 *		*P = 0.238 *	
NNE (dB)	−5.41 ± 1.18	−5.92 ± 1.08	−5.6 ± 1.44	−6.05 ± 1.42
Student's *t*-test	*P = 0.127 *		*P = 0.75 *	

*P < 0.05.
